# The heterogeneity of Covid-19 learning loss across Italian primary and middle schools

**DOI:** 10.1016/j.econedurev.2023.102435

**Published:** 2023-08

**Authors:** Alice Bertoletti, Marta Cannistrà, Mara Soncin, Tommaso Agasisti

**Affiliations:** a Joint Research Centre, Seville 41092, Spain; b School of Management, Politecnico di Milano, Milan 20156, Italy

**Keywords:** Covid-19, Learning loss, Student achievement, Teaching practices

## Abstract

This paper investigates the heterogeneous impact of school closures during Covid-19 pandemic in Italy on academic performance across different schools, grades, subjects and groups of students. Our analysis utilises an innovative dataset that combines administrative data on standardised tests in grades 5 and 8 with a specifically-designed survey that collects information about teachers’ practices between February and June 2020. Firstly, by employing a multilevel (mixed-effects) model, we estimate the extent of learning loss and examine its variability across schools, for students in primary and middle levels during the school year 2020/21. The findings confirm that learning loss has been considerable (between 0.05 and 0.27 SD) although heterogeneity across disciplines and grades exists – higher in English in grade 5, and in mathematics and reading in grade 8. Secondly, as a main contribution of the paper, we explore the mechanisms behind the substantial differences observed across schools, which can be explained by the ability of teachers in using digital tools and evaluating their students, as well as by the leadership role exerted by school principals.

## 1 Introduction

The Covid-19 outbreak generated an unprecedented situation for all the educational systems in the world, forcing schools to close and deliver education remotely. Italy was the first Western country facing the emergency, with one of the longest school closures in Europe – from the end of February 2020 until the start of the new school year (September 2020). During this period, Italian schools had to drastically readapt their systems and teaching modality to a remote learning framework. This process has been particularly challenging for Italian schools, which were less equipped to provide remote teaching compared to other European countries (OECD, 2018). In 2020, Italy reported one of the lowest value of Digital Economy and Society Index, ranking 25th among all 28 EU countries (European Commission, 2020). Similarly, TALIS data reveal that Italian teachers generally had little experience in the use of digital technology and digital learning at school (OECD, 2018).

The length of the school closure and the characteristics of the educational system in the pre-pandemic period make Italy a relevant case of study to understand the effects of Covid-19-related school closures on educational results. To date, there is little quantitative analysis on the effect of Covid-19 school closure on the learning loss of Italian students. Instead, most of the literature focuses on the Northern European countries, which provided rich and high-quality data on students’ standardised tests. On the other hand, these studies give information only on the best-case scenarios (these countries experienced shorter school closures and showed a high level of digital skills and resources also before the pandemic, see Engzell, Frey, & Verhagen, 2021), without being informative on how Covid-19 disruption had affected students in less-prepared European countries.

The term “learning loss” is commonly used in the literature to describe declines in student knowledge and skills (Pier et al., 2021). Regular assessment on learning provides insights into learning growth over time. There is a learning loss when educational progress does not occur at the same rate at which it has historically compared to previous years (Donnelly & Patrinos, 2021).

Extant research is still lacking in providing convincing evidence regarding the mechanisms contributing to differences in learning loss (or gain) across schools. While empirical studies mainly focus on estimating the average effect of Covid-19 school closure on the standardised test scores (see Engzell, Frey, & Verhagen, 2021; Maldonado & De Witte, 2022; Hevia et al., 2022), the results can significantly vary among schools (see Arenas & Gotazar, 2022; Engzell, Frey, & Verhagen, 2021) and subjects (see Schult, Mahler, Fauth, & Lindner, 2022; Arenas & Gotazar, 2022). Although this heterogeneity suggests that school-level and teacher-level characteristics play a relevant role in mitigating the effect of the school closure, these channels remain unexplored in the existing literature (Sternadel, 2021).

This paper addresses these gaps by analysing the heterogeneity of learning loss among Italian students and schools and providing separate results for grades (i.e., grades 5 and 8) and subjects (i.e., reading, mathematics, English). We examine the following research questions:

1 What is the extent of learning loss for Italian students due to the Covid-19 emergency?
2 How does learning loss vary among Italian schools?
3 How do different school characteristics and remote teaching practices influence the learning loss experienced by of Italian students?

The empirical analyses are based on an original dataset we have built by linking the data on INVALSI (National Evaluation Committee for Education) standardised test scores of grade-5 and grade-8 students in 2021 with an ad-hoc survey developed together with INVALSI in 2020. By comparing the Covid-19 cohort with pre-pandemic cohorts of students, we estimate the learning loss of grade-5 and grade-8 pupils in mathematics, reading and English achievement. In a first step, we employ a multilevel (mixed) regression model to estimate the learning loss at school level and explore its heterogeneity among different institutions. In this second step of the empirical work, we examine the relationships between school-level learning loss and some key characteristics of schools and teaching practices during the school closure. The results provide useful insights for understanding the average extent and between-school variation of the learning loss of Italian students and the main factors associated with them, thus contributing to the understanding of the mechanisms behind the observed Covid-19 impact.

To anticipate our main results, Italian students show a substantial learning loss one year after the beginning of the pandemic, with remarkable differences across grades and subjects. In primary school, pupils particularly suffered in English, with a learning gap achieving 0.27 standard deviations (SD). By contrast, in middle school we find larger gaps in mathematics (0.16 SD) and reading (0.08 SD). An important finding of this work is the existence of substantial differences across schools, especially in primary education. This between-school heterogeneity is mainly associated with the way teachers employed digital tools and assessed their students, as well as by the leadership role exerted by school principals during the Covid-19 school closure.

The remainder of the paper is organised as follows: Section 2 summarises the relevant international literature on the learning loss resulting from school closures due to Covid-19, while Section 3 presents the examined data and the survey design. Section 4 describes the methodological strategy we adopt. Then, the results and a final discussion on policy implications are presented in Sections 5 and 6, respectively.

## 2 Academic background

### 2.1 Literature on the effect of Covid-19 school closure on student achievement

Two years after the start of the Covid-19 emergency, the literature offers several studies analysing the effect of Covid-19 school closure on educational outcomes, such as enrolments (Chatterji & Li, 2021), expectations (Rodríguez-Planas, 2022), educational gender gap (Bertoletti et al., 2023) etc. This paper focuses on the effect of emergency on students’ standardised test scores. Concerning this topic, the meta-analyses available in the literature provide evidence of an average negative effect of the school closure on student achievement, equal to −0.14 standard deviation in Betthäuser, Bach-Mortensen, and Engzell (2023), −0.17 standard deviation in Patrinos et al. (2022), and −0.19 standard deviation in Di Pietro (2023). However, the effect was very heterogeneous across countries, subjects and grades. In the Netherlands, Engzell, Frey, and Verhagen (2021) provide evidence of a learning loss of about 3 percentile points or 0.08 SD in the test results of primary-school students. A learning deficit for Dutch kids due to the effect of school closure is also confirmed by the paper of Haelermans et al. (2022). The authors find a consistent learning loss between grades 2 and 5 by analysing the standardised test scores in reading and mathematics in 2020. In particular, the highest values of learning loss is found for mathematics achievement in higher grades. Maldonado and De Witte (2022) examine the 2020 standardised tests of Flemish students in the last year of primary school. The authors report that students of the 2020 cohort experienced significant learning losses in all tested subjects, with a decrease in school averages of mathematics scores of 0.19 SD and Dutch scores of 0.29 SD compared to the previous cohort. Tomasik et al. (2021) have investigated the case of Switzerland, where the Covid-19 school closure lasted only eight weeks. While the results show that secondary-school students did not experience a significant learning loss, primary-education pupils appear to have decreased their achievement by around 0.2 SD. In Norway, Skar, Graham, and Huebner (2022) provide evidence that grade-1 students in the school year 2019/20 reported lower reading performance compared to their peers before the Covid-19 emergency, with a learning loss of 0.24 SD.

The existence of a learning loss is not confirmed across all countries and subjects, especially when a longer time perspective is considered. In a follow-up of the work of Maldonado and De Witte (2020), Gambi and De Witte (2021) find that, after one year from the pandemic, Flemish students are still showing a learning loss in their achievement, but with signals of recovering compared to the previous year. Students stopped increasing their learning deficit in mathematics and French, whereas they started to improve their social science results. Only for reading, the standardised test scores continued to decrease in 2021. On the other hand, Birkelund and Karlson (2021) show that primary-school students in Denmark reported a learning gain in reading in 2021 of about 5 percentage points. Similarly, the work by Schult et al. (2022) shows that fifth graders in Baden-Württemberg, Germany, reported slightly higher scores in reading in 2020 compared to the previous year (i.e., 0.05 SD). Regarding reading achievement, non-significant learning losses were also found for students in grades 4 and 8 in the Basque country (Arenas & Gotazar, 2022), for grade 3 and 8 students in the USA (Lewis et al., 2021), and in Australia, among primary school students (Gore et al., 2021).

As reported above, most of the literature related to European countries is mainly focused on northern regions, but little is known about countries with longer school closures and lower levels of digital skills and resources. In this sense, Hevia et al. (2022) provide an interesting example by analysing the student learning loss in Mexico, where schools closed for 48 weeks. Their results suggest a high negative impact of the pandemic on student achievement, who reported an estimated learning loss of around 0.4 SD in reading and 0.7 SD in mathematics (Hevia et al., 2022). Similarly, Lichand, Doria, Leal-Neto, and Fernandes (2022) find a learning loss of 0.3 SD for secondary school students in Brazil. Finally, one of the highest learning loss estimated in the literature is reported by the paper of Ardington et al. (2021), which shows a decrease of 0.7 SD in the reading performance of South-African primary students.

Despite Italy is one of the European countries that have been most affected by the Covid-19 emergency (UNESCO, 2020), quantitative evidence on the effect of school closure is still limited for Italian students. The recent paper by Contini et al. (2022) has provided evidence on the learning loss in mathematics of grade-3 students in Turin. Although the results cannot be generalised to the entire country, their findings show that, on average, the achievement in mathematics has decreased by 0.19 SD (a result which is coherent with other studies in different countries). In line with this result, Borgonovi and Ferrara (2023) find that, on average, the scores of secondary school students (grade 8) belonging to the Covid cohort are 0.17 SD and 0.08 SD lower in mathematics and reading, respectively. Interestingly, findings for primary students (grade 5) highlights a learning loss of 0.13 SD in mathematics, while a statistically significant learning gain of 0.06 SD in reading. The higher magnitude of learning loss for lower secondary school students can be explained by the lower number of days of school closure experienced by primary schools. Further, authors investigate the achievement gap by SES, gender and previous performance. They report how SES disparities do not increase, while gender gap tends to reduce. Finally, previously middle performers are those who suffered the most the Covid-19 impact. The baseline results are confirmed by Bazoli et al. (2022), who estimate the magnitude of the learning loss for grades 5, 8 and 13. The authors confirm that the higher the grade attended, the more severe the learning loss, as 5-graders even report a statistically significant positive effect in reading. Besides, they report that the effect does not vary regardless the socio-economic stratum of the student.

The present work aims at contributing to the stream of literature on learning loss, analysing the Italian context from a perspective that is doubly innovative: first, beyond reading and mathematics, the Covid-19 effect on English achievement is explored. Second, we estimate the heterogeneity in Covid-19 effect among schools and we link this to teachers’ remote digital practices, to explain potential mechanisms behind students’ learning loss.

### 2.2 Conceptual framework

In addition to the estimation of learning loss due to the pandemic period, it is relevant to understand the factors that potentially account for this gap. The conceptualisation of subsequent analysis goes through a framework's definition. The literature suggests (see Di Pietro et al., 2020) that learning loss’ causes may be disentangled into two main families of determinants: school and individual (family and students) factors. It is worth noting that they are not necessarily unrelated – on the contrary, it is highly likely that they are interconnected, influencing each other.

The role played by schools during the emergency is evident from few academic works. The results by Engzell, Frey, and Verhagen (2021) reveal a high variability in school-level learning loss. Some schools report a learning loss of 10 percentiles or more, whereas other institutions show a small educational gain. Besides, by analysing the standardised test scores of the Basque region in Spain, Arenas and Gotazar (2022) find that the differences in learning loss can be largely explained by school-level factors. These contributions highlight the importance of school-level characteristics, such as the school's ability to react to the emergency, the teachers’ readiness, the resources and the methodologies applied to remote learning (see, Di Pietro et al., 2020). However, the motivations behind between-school variability in learning loss remains unexplored in the empirical literature. On the other hand, more qualitative studies seem to suggest that the reaction to school closure has been heterogeneous across institutions. According to Giovannella et al. (2020), Italian schools with previous experience in digital learning demonstrate to be readier to respond to the pandemic disruption. In general, the most relevant criticalities were the existence of digital barriers among teachers and students and an inefficient organisation of the school system (Carretero et al., 2021; SIRD, 2020). The replication of traditional teaching practices in an online learning environment revealed significant criticalities due to the unsuitability of these tools for the new schooling modalities (Carretero et al., 2021). At the same time, some Italian schools were able to implement innovations and positive experiences to improve their education experience and help their students during the period of school closures (Agasisti & Di Blas, 2021). More recently, Bertoletti et al. (2023) report that a broader use of digital tools for teaching was associated with higher standardised scores of Italian students (INVALSI).

On the other side, academic literature is distinguishing three main individual factors influencing performance of students: family background, socio-emotional skills and previous competencies - with the former having a strong influence on the latter (Hanushek, 1979). Despite previous studies underline how the pandemic exacerbated educational inequality (Betthäuser, Bach-Mortensen, & Engzell, 2023), some others report little or no evidence across students’ socioeconomic status (Arenas & Gortazar, 2022; Birkelund & Karlson, 2021). However, Engzell, Frey, and Verhagen (2021) underlines how for less-educated households, the size of the learning loss is up to 60% larger than in the general population. This evidence shows that learning loss was particularly pronounced for students from disadvantaged families, confirming the fears held by many that school closures would cause socioeconomic gaps to widen. Also, Contini, Di Tommaso, Muratori, Piazzalunga, and Schiavon (2021) find that students with low-educated parents face higher learning loss. Linked to the socioeconomic status of the family, those students are likely to be disadvantaged in their access and ability to use digital learning technology, the quality of their home learning environment, and the learning support they receive from teachers and parents (Goudeau et al., 2021; Van de Werfhorst, 2021). Secondly, the socioemotional skills can play a critical role in explaining the learning loss during the pandemic events (see, for instance, Mushquash & Grassia, 2022). The disruptive switch from face-to-face to online classes, fears of contagion and social distancing had a harmful effect on students’ well-being. Arenas and Gortazar (2022) find learning losses linked to socioemotional well-being deterioration during the pandemic, suggesting strong complementarities between the two. However, as underlined by Shanahan et al. (2022), the measurement of learning loss solely through test scores fails to consider important aspects such as children's psychosocial development and the broader societal costs associated with declines in productivity or increased pressure on parents. Then, despite the expected effects these factors could play on assessing student performance, socioemotional skills are rarely included in the empirical models estimating the learning loss (see Betthäuser, Bach-Mortensen, & Engzell, 2023; Patrinos et al., 2022).

The last set of individual-level determinants relates students’ previous competences. It is well known that education is a cumulative process (Cunha & Heckman, 2007), where previous competences influence the subsequent process of skills formation. Hence, the investigation of the way in which previous performance in estimating learning loss is an interesting area of analysis.

Notwithstanding, recent analyses (Gambi & De Witte, 2021) suggest that the highest decline in test scores belongs to the best-performing students, while low performing students seem to have slightly improved (though insignificantly, in their work). The investigation of the association between previous achievement and learning during the pandemic is object of the only Italian study in the literature. Contini et al. (2021) find that high-performing children from disadvantaged backgrounds faced an important learning loss due to school closures.

Beyond the general assessment of learning loss, this paper aims at contributing to the existing academic literature by offering novel and comprehensive evidence on the heterogeneity that Covid-19 disruption had on student performance, both at student-level and school-level (with the latter being particularly understudied in the literature). Most importantly, the research aims at exploring the mechanisms leading to the significant differences in learning loss of students and schools.

## 3 Data

The first step of the analysis is based on data from INVALSI database. Data refers to a national representative sample of 508 schools in grade 5 (primary schools) and 390 schools in grade 8 (middle schools), selected by INVALSI.^2^ The primary focus of the analysis is on the cohort of students enrolled in the school year 2020/21, which took the INVALSI test in May 2021. These students represent, indeed, the first cohort of students to take the INVALSI test after the Covid-19 disruption – since the test was suspended due to the emergency in 2020. In this vein, this paper estimates the effects of Covid-19 on learning with a one-year lag, without estimating the short-term, immediate impact in the first academic year due to lack of available data. In the school year 2020/21, data refer to 17,159 students in grade 5 and 14,391 students in grade 8. In addition, we compare the Covid-19 cohort with a control group of 75,884 observations– which consists of the entire population of students in the 2018/19 cohort attending the same grades and schools.^3^

INVALSI data provide information on the standardised test scores of the students in reading, mathematics and English (divided into reading and listening). The test scores are built to have a conventional national mean of 200 and a standard deviation of 40. However, to support the interpretation of the results and the comparison across models, the values have been standardised to a mean of zero and a standard deviation of one, in each grade. The psychometric design of the INVALSI tests has undergone changes over time. Specifically, the tests given to primary school children can be compared reliably across different academic years starting from 2018 to 19, while for secondary school children, the tests can be compared reliably from 2017 to 18 onwards. Given that our analysis covers both primary and secondary schools, we can only use a single year-cohort as a control prior to the Covid-19 outbreak (i.e., the 2018/19 cohort).

**Fig. 1:**
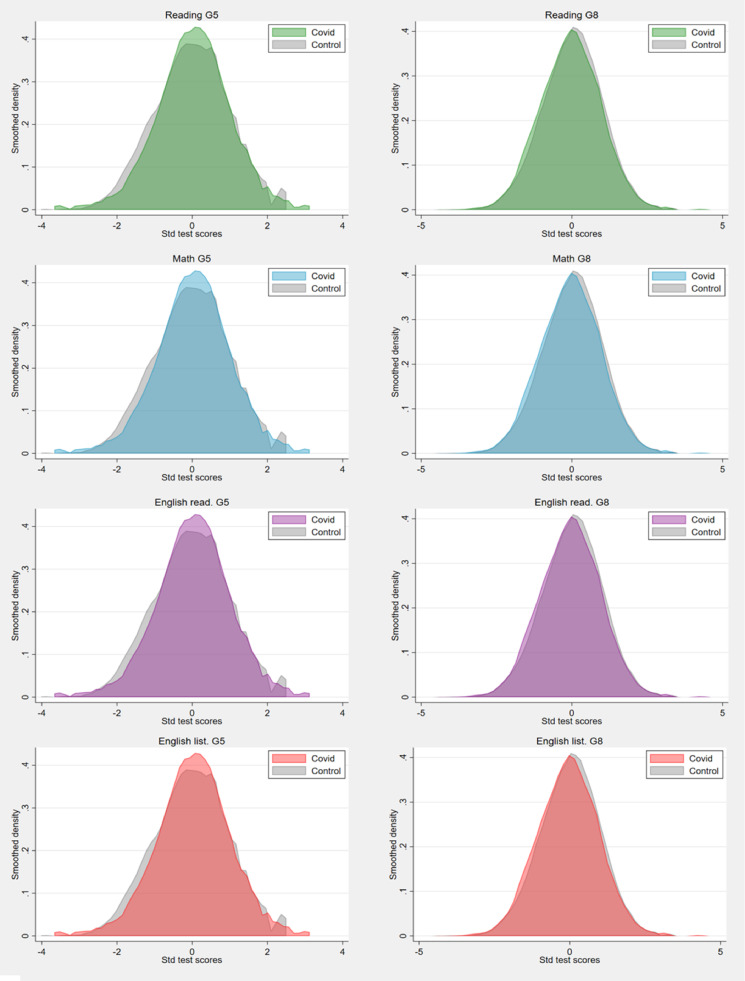
Kernel density plots – Standardised test scores. (For interpretation of the references to colour in this figure legend, the reader is referred to the web version of this article.)

Note: Plots displaying Kernel density of the standardised test scores in reading, math and English per grade. The first column of the figure refers to grade-5 (G5) students, whereas the second one to grade-8 (G8) students. The distributions are reported without propensity score weights.

The distribution of the test scores (described by Kernel densities), divided into treated group (2020/21 cohort) and control group (2018/19 cohort), are reported in Fig. 1. The plots show a marked difference between the distribution of pre-Covid-19 and post-Covid-19 scores in grade 5. Compared to the control group, student achievement in 2019/20 is more distributed around the mean, while fewer observations are present in the tails. Conversely, in grade 8 the Kernel density plots of the control cohort and Covid-19 cohort are more similar.

INVALSI also provides information on students’ characteristics, which have been used as statistical controls in the empirical analyses. In particular, we take into account the gender of the students (female), their nationality (foreign), the socioeconomic status of their family^4^ (ESCS_stud), if at least one of their parents has a higher education degree (parent_he), if students attended kindergarten (kindergarten), and if they postponed their entrance in the educational system (postponed). Among the personal characteristics, we do not have access to the previous performance of the students (in previous grades). Nevertheless, this information is partially modelled by the socioeconomic status, given its high correlation with student performance found in the Italian context (see INVALSI, 2019a). The empirical models also include class/school level information, i.e. the average socioeconomic status of the class (ESCS_class) and the geographical areas (north, south and centre) of the school attended by students. The descriptive statistics for these variables are reported in Tables A1 (for grade 5) and A2 (for grade 8). The share of girls and boys is generally equal in the samples. Most of the students have attended kindergarten and regularly entered into the educational system without postponements. Around one-third of the students have at least one parent with a higher education degree, while not-Italian students are around 10% of the sample. The mean of the students’ socioeconomic status is slightly higher than the national average (between 4% and 14% higher), and the same is found for the ESCS at the class level. Finally, the geographical distribution of the pupils is in line with the population density in the three Italian areas: around 43% in the north, 20% in the centre, and 37% in the south. Tables A1 and A2 (in the Appendix) indicate a significant difference between the control group and the Covid-19 cohort in terms of students’ characteristics (except for gender and foreign). For this reason, as illustrated in the next section, we balance the two samples by employing propensity score weights.

In the second step of the analysis, INVALSI data are matched with the information gathered through an original questionnaire we administered to Italian teachers between July 2020 and September 2020. The survey refers to the same representative sample for which we analyse INVALSI data. However, since the survey was administered one year before the INVALSI test, the questionnaires were sent out to teachers of reading, mathematics and English in grade 4 (primary school) and grade 7 (middle school). In this way, we can match the information on how teachers conducted their online classes in 2020 with the student test scores one year later. The survey response rate ranges between 24% (grade 5) and 31% (grade 8) of the total sample. For this reason, the second-step analysis refers to a subsample of schools nested within the national representative sample. More specifically, in grade 5, the subsample refers to 186 schools (in detail, 134 schools for teachers of reading, 146 for teachers of mathematics and 139 for teachers of English), while there are 185 schools in grade 8 (in detail, 139, 138 and 143 schools for teachers of reading, mathematics and English respectively) .

To evaluate the representativeness of the survey sample, Tables A3 and A4 compare the descriptive statistics of average student characteristics between respondents and non-respondent schools using t-tests (see the Appendix). The analyses indicate differences between the two samples for both grades. Respondent schools have a significantly higher percentage of foreign students, particularly in primary education with 14.2% compared to 10.9% in non-respondent schools. However, the average socioeconomic status of schools is not significantly different between respondent and non-respondent schools at both levels. The average pre-pandemic results are similar between respondent and non-respondent schools, except for mathematics at grade 8, where non-respondent schools report higher achievements on average. Moreover, the differences between respondent and non-respondent schools are mostly related to geographical distribution, with northern schools having a higher response rate than southern schools for both grades.

To address the imbalance between the two samples, we employ Inverse Probability Treatment Weights in the second-step regression analysis (see Section 4.2). Although some differences exist between the two samples, the statistics reported in Tables A3 and A4 do not indicate the presence of clear self-selection bias between respondent and non-respondent schools (i.e., respondent teachers are not necessarily representative of better-performing schools). Therefore, since we use weights to balance the characteristics of the two samples, the results presented in the subsequent section may be considered to have an adequate level of external validity.

The survey collects quite detailed information on how teachers conducted their online classes during the lockdown. More specifically, five sections of questions compose the surveys: technologies supporting (i) synchronous teaching and (ii) asynchronous teaching, (iii) modalities of students’ assessment, (iv) support from school principals, (v) frequency and duration of online classes. It is worth noting that, for the primary and middle levels, school closure exclusively concerned the period between March 2020 and June 2020. Therefore, the information we have collected through the survey can be considered representative of the overall period of remote teaching.

Table 1 describes all the variables coming from the survey. The first six indicators measure the digital tools employed by teachers to support their synchronous and asynchronous classes and the methods used to assess their students. As described in Table 2, most of the teachers used to share text material with the students after the classes, while videos were used by almost half of the teachers during synchronous remote lessons. Apps and survey tools were less frequently used by teachers and appear to be more specific to the grade and subject in which they were employed. Learning apps were more likely to be used by English teachers, while surveys were more frequently utilised in middle schools. Teachers assessed students mainly by evaluating their homework and their attendance during the online classes, whereas oral tests were less frequent. The questionnaire also provides information on the weekly hours of synchronous lessons per teacher. Grade-8 teachers and, in particular, teachers of reading reported the highest number of hours per week (8.4 h/week). Also, around one-third of the middle-school teachers started online classes within one week after school closure, whereas the share was lower in primary schools (i.e., 20%). Finally, we consider if teachers received clear information and support from the school principal on how to organise their remote classes. One-third of the teachers reported being satisfied, without significant difference across subjects or grades (see Table 2).

**Table 1:** Survey about the teaching practices during Covid-19 school closure: selected variables.

Variable	Description	Type
sync_survey	If teacher frequently used online surveys during synchronous online classes in 2020	dummy
sync_video	If teacher frequently used videos during synchronous online classes in 2020	dummy
async_text	If teacher frequently shared text materials to support asynchronous online classes in 2020	dummy
async_app	If teacher frequently online apps materials to support asynchronous online classes in 2020	dummy
assess_attendance	If teacher frequently used students' attendance for assessing them during 2020 school closure	dummy
assess_homework	If teacher frequently used students' homework for assessing them during 2020 school closure	dummy
assess_oral	If teacher frequently used oral tests for assessing students during 2020 school closure	dummy
SP_guidance	If the teacher received clear information and support from School Principal during the 2020 school closure	dummy
quick_start	Start of synchronous digital teaching after 2020 school closure, immediately or after 1 week.	dummy
h_synchronous	Hours of digital teaching by week, on average	[0; 40] range

Note: statistics refer to 1382 observations. Teachers’ responses are aggregated at school level, separately per grade and subject. On average, there are 1.8 subject-specific teachers per school.

**Table 2:** Survey about the teaching practices during Covid-19 school closure: descriptive statistics of selected variables.

Variable	Total	Reading G5	Reading G8	Mathematics G5	Mathematics G8	English G5	English G8
sync_survey	0.102	0.074	0.115	0.087	0.151	0.082	0.103
	−0.246	−0.211	−0.25	−0.241	−0.284	−0.217	−0.261
sync_video	0.478	0.496	0.491	0.487	0.471	0.473	0.453
	−0.408	−0.413	−0.402	−0.434	−0.405	−0.401	−0.399
async_text	0.776	0.730	0.826	0.765	0.849	0.691	0.803
	−0.331	−0.343	−0.293	−0.348	−0.268	−0.365	−0.334
async_app	0.366	0.374	0.317	0.348	0.345	0.407	0.404
	−0.38	−0.39	−0.331	−0.384	−0.377	−0.391	−0.397
assess_attendance	0.847	0.822	0.917	0.783	0.916	0.745	0.91
	−0.319	−0.341	−0.217	−0.362	−0.241	−0.397	−0.256
assess_homework	0.857	0.783	0.950	0.922	0.895	0.807	0.789
	−0.306	−0.364	−0.177	−0.231	−0.253	−0.338	−0.375
assess_oral	0.491	0.504	0.514	0.500	0.437	0.436	0.565
	−0.453	−0.457	−0.447	−0.478	−0.414	−0.466	−0.447
SP_guidance	0.343	0.322	0.344	0.391	0.361	0.292	0.35
	−0.384	−0.381	−0.366	−0.407	−0.393	−0.356	−0.395
quick_start	0.268	0.183	0.376	0.170	0.374	0.181	0.336
	−0.389	−0.332	−0.414	−0.343	−0.411	−0.329	−0.43
h_synchronous	7.150	7.040	8.400	6.920	7.820	5.510	7.340
	−4.986	−5.377	−4.554	−6.023	−3.851	−5.168	−4.141
N. observations	1382	230	218	230	238	243	223

Note: The values of variables represent the proportion of teachers who positively respond to the questions (see Table 1). Standard errors in parentheses. G5 indicates grade 5 (primary schools), and G8 grade 8 (middle schools). Teachers’ responses are aggregated at school level, separately per grade and subject. On average, there are 1.8 subject-specific teachers per school.

## 4 Empirical strategy

The empirical strategy of this paper is articulated in two steps. In the first step, we estimate general (system-level) and specific (school-level) learning loss due to Covid-19 school closures. In the second step, we estimate the relationship between school-level learning loss and school-level characteristics.

### 4.1 First step

The first step of the empirical analysis aims at estimating the learning loss associated with the Covid-19 school closure. Following this purpose, we analyse INVALSI standardised test scores, which measure the mathematics, reading and English achievement at the end of the school year 2020/2021. Since the INVALSI test was suspended in 2019/2020, we can estimate the effect of Covid-19 school closure only one year after Covid-19 school closure. We compare the INVALSI scores of students in the school year 2020/21 (the treatment group) with the ones obtained by two pre-pandemic cohorts of students (control group) attending the same grades and schools. Thus, the general model estimating the impact of Covid-19 on student achievement is described by the following equation:

(1) $$Y_{i}=\beta_{0}+\beta_{1}C_{i}+\beta_{2}X_{i}+e_{i}\;\forall \;b\;and\;g$$

where *Y*i is a standardised test score of a student i; *C*k is a dummy variable equal to 1 if the student is in the Covid cohort k, 0 otherwise; *Xi* is the vector of students’ characteristics and sociodemographic variables described in Section 3 (i.e., migratory background, parental education, kindergarten attendance, postponed entrance in education, socioeconomic status of parents and class average, geographical area of the school); *ei* are stochastic errors normally distributed and clustered at the class level. Model (1) is estimated separately by grade g (i.e., grades 5 and 8) and subject b (i.e., reading, mathematics, English reading and English listening). The coefficient of interest is *β*1, which captures the causal effect of being part of the Covid-19 cohort, rather than the pre-Covid-19 cohorts, on education achievement in a given subject and grade. Thus, finding a negative value of *β*1 would suggest the existence of an average learning loss of the students (a positive coefficient indicates, instead, a learning gain). As described in the previous section, the outcome variable, Y, is standardised to have a mean of zero and a standard deviation of one in their respective grade and subject. The standardisation has been performed jointly considering all the years, in order to compare the different cohorts of students.

**Fig. A1:**
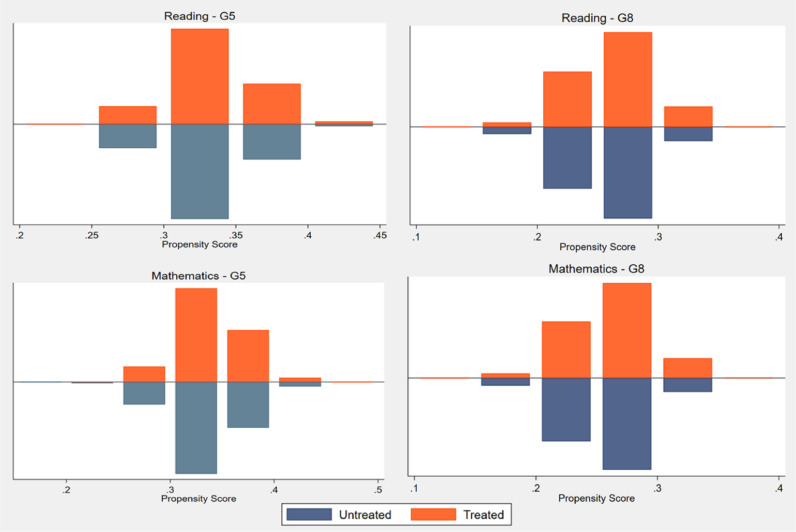
Propensity scores for balancing Treated and Control groups - Reading and Mathematics. (For interpretation of the references to colour in this figure legend, the reader is referred to the web version of this article.)

**Fig. A2:**
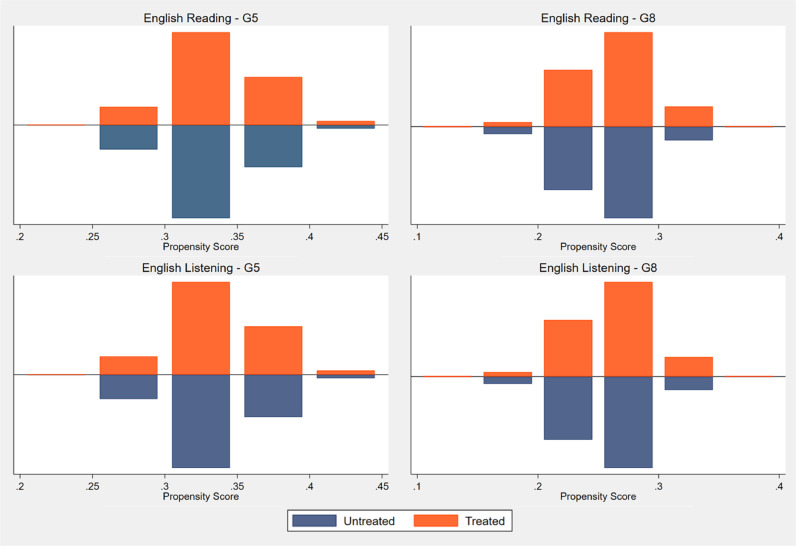
Propensity scores for balancing Treated and Control groups - English. (For interpretation of the references to colour in this figure legend, the reader is referred to the web version of this article.)

As shown in Section 3, the students in the treatment group (2020/21 cohort) and the ones in the control group (2016/17 and 2018/19 cohorts) present several differences in their characteristics. Thus, we balance the two samples to improve the comparison between the two groups of students. Following Engzell, Frey, and Verhagen (2021), we compute the propensity scores of belonging to the treatment group and employ them as weights while estimating model (1). The propensity scores are computed following the Full Mahalanobis matching, through which we balance the two groups over the covariates of vector *Xik* in (1). More specifically, the propensity scores are calculated based on the following equation:

(2) $$1/N1*\text{Var}\left(Y\;|\;\text{DM}=1\right)+\;\sum \left(w_{j}^{2};\;i\;\text{in}\;\text{DM}=0\right)/N1^{2}*\text{Var}\left(Y\;|\;\text{DM}=0\right)$$

where N1 is the number of matched treated, DM=1 denotes the matched treated, DM=0 the matched controls, and w_j_ is the weight given to control j. Figs. A1 and A2 in the Appendix display the distribution of the propensity scores employed for balancing the sample.

The coefficient *β*_1_ in model (1) provides an estimation of the average effect of Covid-19 school closure. However, to explore the second research question, we need to estimate a school-level effect that can highlight differences in reacting to the pandemic among institutions. To this aim, we estimate a multilevel (mixed) model, in which the coefficient of interest (*β*_1_) is allowed to vary among schools. Thus, we modify Eq. (1) into the following model:

(3) $$Y_{is}=\;\beta_{0}+\;\beta_{1}X_{is}+\alpha_{1s}C_{is}+\alpha_{0s}+\;e_{is}\;\forall \;b\;and\;g$$

The last part of the equation (terms in bold) represents the random part of the model that can vary across schools s. The effect of Covid-19 school closure is now described by the vector of coefficients α_1s_, which includes different estimated values for each school. Vector $\alpha_{0s}$ is instead the random intercept and captures the school value-added on student achievement. These values are not related to the Covid-19 disruption since is constant over the treated and control cohorts. Thus, $\alpha_{{}_{1s}}$ can provide information on the specific impact of Covid-19 disruption for each school, net of the average school value-added. It is important to clarifying that, in this paper, we do not aim to describe and analyse the value added. Finally, the controls $X_{is}$ are constant over schools and represent the fixed part of the model. Through this empirical approach, our findings do not confound the effectiveness of schools with their specific role in affecting/mitigating the Covid-19 school closure on learning loss.

### 4.2 Second step

In the second step of the analysis, we aim at explaining the variation of the school-level learning loss by means of the ad hoc survey variables on how teachers conducted their online classes during school closure. To this aim, we regress the variables described in Table 1 over the estimated random value of the Covid-19 effect (i.e., vectors $\alpha_{1s}$ in Eq. (3)), found in the previous step. The model is described by the following equation:

(4) $$\hat{\alpha_{1s}}=\;\gamma_{0}+\;\gamma_{1}Z_{s}+\;u_{s}\;\forall \;b\;and\;g$$

where $\hat{\alpha_{1s}}$ is the estimated effect of Covid-19 on students in grade g and subject b in school s; $Z_{s}$ is the vector of the variables associated with the teachers of subject b in grade g in school s; $u_{s}$ are stochastic errors. The vector $Z_{s}$ is computed by aggregating the responses of the teachers of subject b and grade g at school level. On average, there are 1.8 responding teachers per school, grade and subject. As for the previous models, Eq. (4) is estimated separately by grade g and subject b.

As described in Section 3, model (4) is estimated on a subsample of schools that is driven by the survey response rate of teachers in the specific grade and subject. However, Tables A3 and A4 show that some statistically significant differences exist between respondent and non-respondent schools in both grades, in terms of students’ characteristics and geographical location. Therefore, to increase the external validity of our results, Inverse Probability Treatment Weighting (IPTW) is applied to the observations available for the survey data. More specifically, the weights are calculated as in Eqs. (5) and (6):

(5) $$W_{s}=\frac{1}{p{\left(X\right)}_{s}}\;\forall \;b\;and\;g$$

(6) $$p{\left(X\right)}_{s}=P(C_{s}=1|X_{s})\;\forall \;b\;and\;g$$

where $W_{s}$ is the vectors of IPTW weights, and $p{\left(X\right)}_{s}$ is the propensity score of a school (s) to be treated (i.e., to have teachers responding to the survey for the specific grade and subject), given a vector of observable characteristics $X_{s}$. More specifically, $X_{s}$ includes the ESCS of the school, the school average results in the specific subject before 2020, the school average percentage of foreign students, and the geographical area of the school (i.e., north, south and centre). The propensity scores are estimated by employing a logit model.

## 5 Results

### 5.1 System-level (average) learning loss

**Fig. 2:**
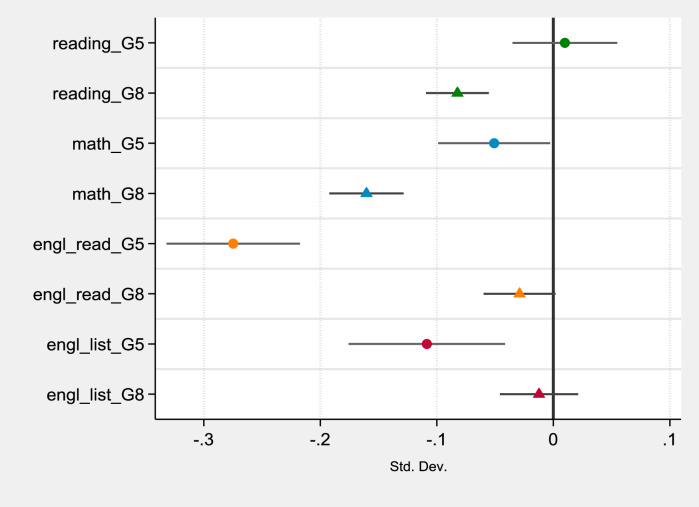
Covid-19 effect on standardised test scores per subject and grade. (For interpretation of the references to colour in this figure legend, the reader is referred to the web version of this article.)

Note: the figure reports the estimates of *COVID* coefficients and the respective confidence intervals of the eight models in Table 4. The subjects are displayed with different colours and grades with different shapes. G5 indicates grade 5 (primary schools), and G8 grade 8 (middle schools).

Based on the model described in (1), we estimate separately by subject and grade the effect of being in the Covid-19 cohort on student achievement (see Table 3). The coefficients of the variable of interest, COVID, indicate the effects of the Covid-19 disruption on INVALSI standardised test scores. The estimates of COVID coefficients are plotted for each subject and grade in Fig. 2, which allows for a direct and synthetic interpretation of the results.

**Table 3:** Learning loss estimates per subject and grade.

	(1)	(2)	(3)	(4)	(5)	(6)	(7)	(8)
Variables	Reading G5	Reading G8	Mathematics G5	Mathematics G8	English reading G5	English reading G8	English listening G5	English listening G8
COVID	**0.011**	−0.085***	−0.052**	−0.163***	−0.275***	−0.029*	−0.109***	**−0.012**
	(0.022)	(0.014)	(0.024)	(0.016)	(0.029)	(0.015)	(0.034)	(0.017)
female	0.186***	0.229***	−0.151***	−0.127***	0.100***	0.192***	0.130***	0.185***
	(0.011)	(0.009)	(0.011)	(0.010)	(0.011)	(0.009)	(0.011)	(0.009)
parent_he	0.217***	0.195***	0.166***	0.196***	0.136***	0.186***	0.119***	0.187***
	(0.017)	(0.013)	(0.017)	(0.013)	(0.018)	(0.013)	(0.018)	(0.013)
ESCS_stud	0.155***	0.245***	0.164***	0.222***	0.122***	0.229***	0.104***	0.205***
	(0.008)	(0.006)	(0.008)	(0.006)	(0.008)	(0.006)	(0.008)	(0.006)
foreign	−0.275***	−0.323***	−0.187***	−0.124***	0.081***	0.111***	0.159***	0.272***
	(0.020)	(0.018)	(0.021)	(0.018)	(0.021)	(0.018)	(0.022)	(0.018)
postponed	−0.464***	−0.411***	−0.366***	−0.428***	−0.288***	−0.407***	−0.145***	−0.286***
	(0.047)	(0.023)	(0.046)	(0.022)	(0.056)	(0.025)	(0.054)	(0.024)
kindergarten	0.121***	0.115***	0.142***	0.106***	0.0517	0.09***	0.0167	0.062**
	(0.040)	(0.024)	(0.049)	(0.025)	(0.042)	(0.026)	(0.049)	(0.024)
ESCS_class	0.097***	0.169***	0.063**	0.196***	0.0486*	0.235***	0.119***	0.284***
	(0.022)	(0.016)	(0.025)	(0.018)	(0.026)	(0.018)	(0.033)	(0.019)
centre	0.051**	−0.082***	0.074**	−0.142***	−0.047	−0.168***	−0.070*	−0.221***
	(0.024)	(0.017)	(0.032)	(0.020)	(0.031)	(0.018)	(0.039)	(0.018)
south	−0.090***	−0.286***	0.0276	−0.460***	−0.112***	−0.435***	−0.239***	−0.610***
	(0.022)	(0.014)	(0.025)	(0.016)	(0.029)	(0.016)	(0.033)	(0.017)
constant	−0.215***	−0.054**	−0.108**	0.206***	−0.0148	−0.0103	0.00337	0.0617**
	(0.042)	(0.025)	(0.051)	(0.028)	(0.046)	(0.027)	(0.053)	(0.026)
Observations	33,601	37,461	34,057	37,420	33,238	37,392	33,222	37,383
R-squared	0.106	0.206	0.079	0.196	0.057	0.204	0.056	0.239

Note: *** *p* < 0.01, ** *p* < 0.05, * *p* < 0.1. Clustered standard errors per class in parentheses. The dependant variables of the eight models are the INVALSI test scores in different grades and subjects. G5 indicates grade 5 (primary schools), and G8 grade 8 (middle schools). The scores are standardised to have a mean of zero and a standard deviation of one in their respective grades and cohorts. Models estimated using propensity score weights.

On average, students in the Covid-19 cohort attending primary education (grade 5) have experienced a significant reduction in their English achievement, equal to −0.11 SD in English listening and −0.27 SD in English reading. For the latter variable, the results show a considerable size of the effect – that is, for instance, more than double the effect found for the socioeconomic status (ESCS_stud). Additionally, the reduction in mathematics performance is statistically significant, although the effect size is limited (i.e. 0.08 SD). On the other hand, the same students did not show a statistically significant change in their reading scores compared to the pre-Covid-19 cohorts.

At middle schools, the estimates indicate a different situation. Students taking the test in 2021 reported a statistically significant decrease in mathematics and reading achievement. The extent of the coefficient is larger for mathematics than for reading scores (with −0.16 SD and −0.08 SD, respectively). Nevertheless, the effect of Covid-19 disruption is not statistically significant for English listening achievement, while it is slightly significant for the English reading scores showing an effect of −0.03 SD.

The control factors are all statistically significant and report directions and sizes of the effects in line with the literature in the Italian context (see, for instance, Agasisti & Falzetti, 2017; Cascella, 2020; INVALSI, 2019b). Girls performed better in reading and English but achieved lower test scores than boys in mathematics. Parental education and the socioeconomic status of students/classes represent the most relevant control for the academic outcomes in all subjects and grades with non-advantaged students (in non-advantaged schools) suffering less the effects of school closures. By contrast, students who have postponed their entrance into the educational system tend to perform worse, whereas attending kindergarten positively affects academic achievement. Students with non-Italian citizenship are associated with lower results in reading and mathematics but, on the contrary, they tend to obtain higher performance in English. Finally, students attending school in northern regions reported higher scores than the ones in central and, especially, southern regions.

**Fig. 3:**
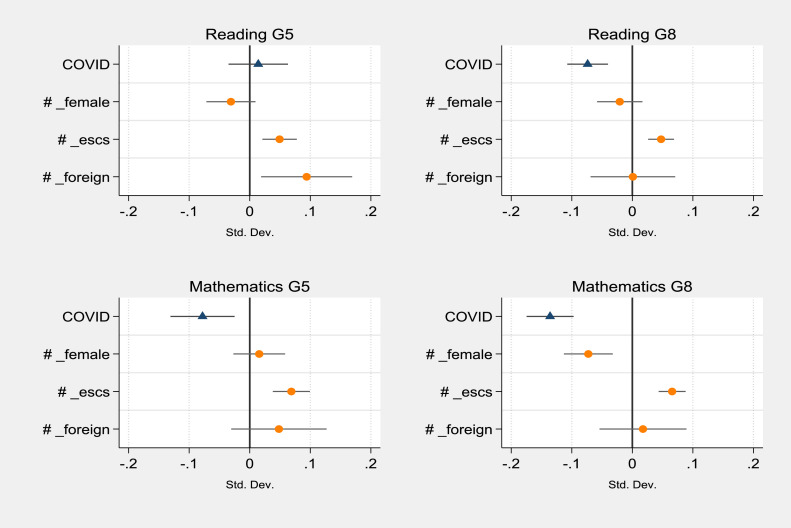
Heterogeneity of Covid-19 effect on different groups of students on standardised test scores in reading and mathematics. (For interpretation of the references to colour in this figure legend, the reader is referred to the web version of this article.)

Note: the figure is based on the results of four regression models on the standardised test score of students in reading and mathematics. Each graph reports the estimated coefficients and the confidence intervals for *COVID* and *COVID* interactions with student gender (female = 1), students’ socioeconomic status (ESCS) and student citizenship (foreign = 1). Each model includes the following controls (not reported in the figure): female, postponed, kindergarten, ESCS_stud, foreign, ESCS_class, and geographical areas. Models estimated using propensity score weights. G5 indicates grade 5 (primary schools), and G8 grade 8 (middle schools).

**Fig. 4:**
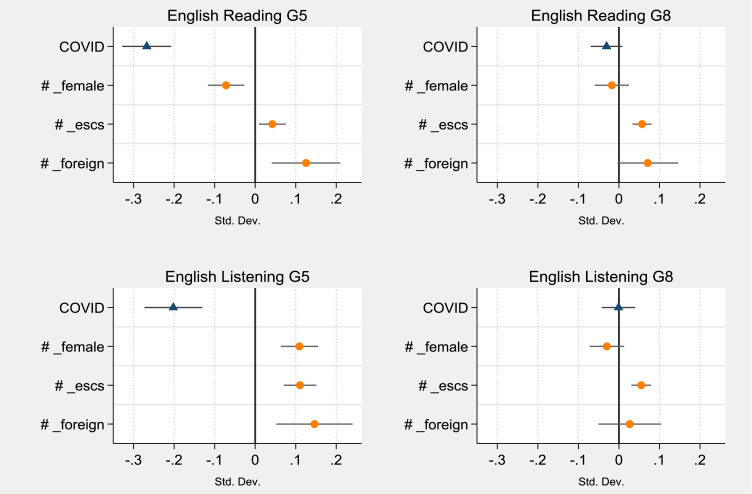
Heterogeneity of Covid-19 effect on different groups of students on standardised test scores in reading and mathematics. (For interpretation of the references to colour in this figure legend, the reader is referred to the web version of this article.)

Note: the figure is based on the results of four regression models on the standardised test score of students in English (reading and listening). Each graph reports the estimated coefficients and the confidence intervals *COVID* and *COVID* interactions with student gender (female = 1), students’ socioeconomic status (ESCS) and student citizenship (foreign = 1). Each model includes the following controls (not reported in the figure): female, postponed, kindergarten, ESCS_stud, foreign, ESCS_class, and geographical areas. Models estimated using propensity score weights. G5 indicates grade 5 (primary schools), and G8 grade 8 (middle schools).

To explore the heterogeneity of the effect of Covid-19 disruption on different groups of students, we replicate the models in Table 3 by adding interactions between the treatment (COVID) and student characteristics (i.e., gender, socioeconomic status and citizenship). The estimates of the coefficients associated with the Covid-19 effect and its interactions are displayed in Figs. 3 and 4 (see Table A5, in the Appendix, for the complete estimates). The interactions with students’ socioeconomic status report positive and statistically significant effects in all grades and subjects, indicating that students from wealthier families are the ones who suffered less learning loss. The result is fully in line with the findings in the literature, which shed light on the socioeconomic inequality of the effect of Covid-19 school closure (see, for instance, Maldonado & De Witte, 2022; Engzell, Frey, & Verhagen, 2021; Gore et al., 2021). Gender interactions are instead characterised by more heterogeneous effects. A statistically significant effect is found for mathematics in grade 8, with a negative coefficient of −0.07 SD, highlighting that not only are girls usually presenting lower levels of mathematics achievement (see Table 4), but they are also the ones most affected by Covid-19. The effect of gender interaction is also significant for English scores in grade 5, but with opposite directions between reading and listening. Compared to boys, female students were less affected by school closure in listening (0.11 SD) but were more harmed in English reading (−0.07 SD). Finally, during school closure, non-Italian students in grade 5 appear to have improved their academic performance in reading and English. The results for English may be explained by a higher likelihood of speaking English at home with their parents compared to Italian students - and they are in line with the general trend that reports higher English achievement for international students (see INVALSI, 2019b). The finding for reading in grade 5 (0.09 SD) is more controversial to interpret. Indeed, even if international students are confirmed to obtain lower results in reading (−0.32 SD in grade 5), the Covid-19 cohort reported higher scores compared to the ones before the lockdown. A possible explanation could be linked to a wider adaptivity of digital learning tools to the personal needs of students (Bando et al., 2017), especially for younger students. Through these tools, non-Italian pupils could have exploited the period of school closure to catch up with their learning gaps in Italian and improve their performance. The available data does not allow further exploring this potential mechanism in detail.

**Table 4:** School-level effect of Covid-19 disruption by type of school.

	(1)	(2)	(3)	(4)	(5)	(6)
Variables	Reading G5	Reading G8	Mathematics G5	Mathematics G8	English G5	English G8
pre-Covid scores	−0.187***	0.019	−0.138**	−0.022	−0.406***	−0.037
	(0.072)	(0.031)	(0.062)	(0.044)	(0.068)	(0.033)
% foreign students	0.024	−0.081	−0.074	−0.178*	0.113	−0.012
	(0.127)	(0.059)	(0.157)	(0.093)	(0.201)	(0.068)
% HE parents	0.236***	0.004	−0.019	0.071	0.278***	0.069
	(0.081)	(0.037)	(0.100)	(0.060)	(0.104)	(0.050)
school size	0.002	−0.000	−0.000	−0.001	0.001	−0.000
	(0.002)	(0.001)	(0.002)	(0.001)	(0.003)	(0.001)
centre	−0.020	0.002	0.074	0.019	−0.035	−0.015
	(0.031)	(0.017)	(0.049)	(0.029)	(0.052)	(0.020)
south	−0.077	−0.021	0.025	−0.061*	−0.196***	−0.070**
	(0.048)	(0.020)	(0.053)	(0.032)	(0.057)	(0.028)
constant	−0.099	−0.014	−0.035	−0.049	−0.170	−0.001
	(0.073)	(0.030)	(0.095)	(0.047)	(0.110)	(0.038)
N. schools	456	336	457	336	452	334
R-squared	0.043	0.013	0.015	0.028	0.115	0.032

Note: *** *p* < 0.01, ** *p* < 0.05, * *p* < 0.1. Robust standard errors in parentheses. The dependant variables of the six regression models are the school-level Covid-19 effects shown in Fig. 5 for the respective grade and subject. North is the reference category for the geographical area. School size is measured by the number of students per school. Pre-Covid-19 scores represent the standardised school mean of the student score of the respective subject and grade in the two years before the pandemic. The ESCS of the school is not included to avoid problems of collinearity with pre-Covid-19 scores. Note that school ESCS is not statistically significant when pre-Covid-19 scores are not included in the models. G5 indicates grade 5 (primary schools), and G8 grade 8 (middle schools). The schools in the sample are all public schools.

### 5.2 Exploring the school-level learning loss

**Fig. 5:**
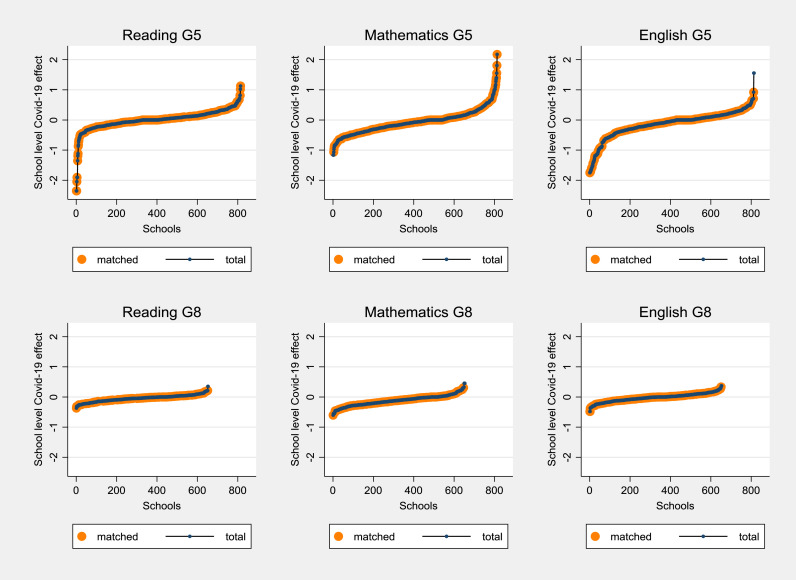
Random effects of Covid-19 at school level per grade and subject. (For interpretation of the references to colour in this figure legend, the reader is referred to the web version of this article.)

Note: the graphs report the school-level effects for the total national sample in blue, whereas the schools in the sample are displayed in orange. G5 indicates grade 5 (primary schools), and G8 grade 8 (middle schools).

Not only is the response to the Covid-19 disruption heterogeneous across groups of students, but it is also different across schools. This finding is revealed by the estimates of the multilevel analysis, which show the between-school variation of the (random) effect of Covid-19 disruption on student test scores (see Fig. 5). Fig. 5 sheds light on how the Covid-19 emergency produced heterogeneous effects across schools, especially in grade 5, whereas less variability exists for grade 8. Based on this evidence, the absence of a system-level learning loss found in the previous section for reading and mathematics in grade 5 is probably led by the wide heterogeneity between schools. While some schools turned this disruption to their advantage and generated an increase in student achievement, other institutions are associated with a large learning loss - with a size that can exceed two standard deviations (see reading grade 5). The findings suggest that primary schools were probably less prepared to deal with the school closure forced by the pandemic, while middle schools were able to respond to the crisis more homogeneously. Indeed, in middle schools, a minimum number of synchronous online classes per week was often settled by the school leader. In contrast, the situation at the primary level was more flexible: given the lower age of the students, online classes were more complicated to organise and the survey data show, indeed, that in some cases synchronous online lessons were not implemented (see Table 1). In other cases, primary schools decided to limit the hours of online classes, leaving the priority to eventual older siblings that needed to use digital devices or internet connection. These potential explanations are in line with the data reported in Table 2, which show that the variability of the hours of the synchronous class is considerably higher in primary than in middle schools.

In Table 4, we describe how the effect of Covid-19 on students’ learning varied across different types of schools. The estimates show that the schools in primary education with higher student performance in the years before the pandemic are the institutions harmed the most by the school closure. More specifically, an increase of 1 SD in the average pre-pandemic results is associated with a learning loss of 0.19 SD in reading, 0.14 SD in mathematics and 0.40 SD in English. On the other hand, the coefficient is no more statistically significant for student achievement in grade 8 – meaning that the magnitude of the learning loss did not vary accordingly to previous results in middle schools. A 10% higher percentage of non-Italian students in the school is associated with 0.02 SD higher learning loss in mathematics in middle education - whereas a 10% increase in the share of students with higher educated parents^5^ is linked to 0.02 SD and 0.03 higher scores in reading and English at primary school. Besides, there is no apparent correlation between school size (measured by the number of enrolled students) and the average learning loss in primary and elementary schools. Finally, geographical differences in the distribution of learning loss across schools are found for English test scores - with southern institutions presenting on average a 0.20 SD (in grade 5) and 0.07 SD (in grade 8) larger learning loss compared to the ones in the north of Italy.

To explore how teaching-related factors are associated with the between-school variability in learning loss, we match the school-level effects of Covid-19 reported in Fig. 5 with the survey data of the same class in the school year 2019/20 (i.e., the second step of the empirical analysis). The teachers' responses have been aggregated at the school level but maintain the separation across subjects and grades. In this way, the school-level estimates of learning loss can be explained exclusively by the information provided by the teachers in the specific subject (see Section 3).

Table 5 reports the estimates of a weighted OLS model on the school-level learning loss for each subject and grade (see Eq. (4) in Section 4.2.). The models include the survey variables collected through the survey and described in Table 1. The estimates associated with synchronous and asynchronous digital tools shed light on the importance of the way through which teachers conducted remote classes, especially for reading and English. The results are heterogeneous across models, suggesting that the digital tools adapted differently to the features of specific grades and subjects. More specifically, using videos to support synchronous class has been effective for reading in grade 5, and this is associated with 0.17 SD higher reading scores/lower learning loss. Employing synchronous surveys and sharing text material with students are linked to positive student achievement in reading in middle education (0.063 SD and 0.057 SD, respectively). Inversely, the use of surveys (like self-evaluation quizzes or tests) has a statistically significant negative effect on the results in reading and English of grade 5 students (−0.14 and −0.39 SD, respectively). In addition, schools where teachers used apps to support asynchronous classes are associated with slightly lower scores/higher learning loss in reading and English in grade 8 (−0.08 SD and −0.10 SD, respectively).

**Table 5:** Teaching-related factors associated to school-level effect of Covid-19 learning loss.

	(1)	(2)	(3)	(4)	(5)	(6)
Variables	Reading G5	Reading G8	Mathematics G5	Mathematics G8	English G5	English G8
sync_video	0.172***	−0.040*	−0.031	−0.041	0.086	−0.004
	(0.053)	(0.022)	(0.052)	(0.034)	(0.076)	(0.026)
sync_survey	−0.141*	0.063**	−0.082	−0.042	−0.394**	0.012
	(0.072)	(0.030)	(0.104)	(0.045)	(0.180)	(0.050)
async_text	0.033	0.057**	0.086	0.030	0.030	0.026
	(0.069)	(0.025)	(0.063)	(0.056)	(0.083)	(0.033)
async_app	0.069	−0.083***	−0.020	0.043	0.030	−0.097***
	(0.059)	(0.022)	(0.058)	(0.033)	(0.075)	(0.029)
assess_homework	−0.075	−0.025	0.024	0.150**	−0.290***	−0.082**
	(0.051)	(0.034)	(0.085)	(0.065)	(0.095)	(0.032)
assess_oral	−0.012	0.030*	0.154***	−0.050	−0.120*	0.000
	(0.050)	(0.018)	(0.059)	(0.039)	(0.067)	(0.024)
assess_attendance	0.086	0.007	0.142**	−0.050	0.138*	−0.028
	(0.058)	(0.032)	(0.063)	(0.038)	(0.078)	(0.039)
SP_guidance	0.011	−0.013	0.013	0.075**	0.041	0.061**
	(0.064)	(0.021)	(0.057)	(0.033)	(0.093)	(0.025)
quick_start	−0.031	0.036**	−0.141*	−0.040	0.300***	0.049*
	(0.064)	(0.018)	(0.082)	(0.033)	(0.090)	(0.027)
h_synchronous	−0.005	0.003*	0.005	0.005	0.010	−0.003
	(0.006)	(0.001)	(0.004)	(0.003)	(0.007)	(0.002)
Controls	yes	yes	yes	yes	yes	yes
Constant	−0.121	−0.043	−0.435***	−0.190**	0.265	0.122*
	(0.181)	(0.055)	(0.123)	(0.092)	(0.202)	(0.067)
Observations	221	205	223	217	235	200
R-squared	0.129	0.175	0.153	0.141	0.258	0.196

Note: *** *p* < 0.01, ** *p* < 0.05, * *p* < 0.1. Standard errors in parentheses. The dependant variables of the six regression models are the school-level Covid-19 effects shown in Fig. 5 for the respective grade and subject. Controls include: school size, the ESCS, the share of foreign students, and the geographical area of the schools. Estimates using Inverse Probability Treatment Weighting (IPTW). G5 indicates grade 5 (primary schools), and G8 grade 8 (middle schools).

One of the most influential factors is, however, the way teachers evaluated their students. Assessing students based on their homework is associated with a larger learning loss/lower result in reading in grade 5 and English in both grades (English grade 5 has the largest size of the coefficient, with a −0.29 SD effect). By contrast, this type of assessment seems to be effective for mathematics achievement in grade 8 (with a 0.15 SD effect). This difference could be related to the diversity of analytical subjects compared to languages, with math exercises being probably more suitable to be employed for evaluation purposes. Nevertheless, this reasoning seems not to hold for younger students. Indeed, in primary school the positive effect of homework evaluation is no longer significant, and higher math scores are instead associated with oral tests and the evaluation of students’ attendance (with a 0.15 SD increase for both types of evaluation).

Support from the school principal, providing clear information on how to conduct remote teaching classes, is associated with higher scores/lower learning loss in math and English in grade 8 (0.07 SD and 0.06 SD, respectively). The result suggests that the support from the school leadership was particularly relevant in secondary education, where teachers were indeed likely to deal with higher organisational complexity. Moreover, a quick start of online classes (within one week after the school closure) positively affected student test scores in reading and English. The effect is specifically large for English in grade 5, where these schools present 0.3 SD lower learning loss compared to the institutions that did not rapidly start online classes. Finally, the weekly number of hours of synchronous classes is, in general, positively correlated to student achievement but without any large statistically significant effect.

## 6 Discussion and conclusion

This paper provides new evidence on the effect of Covid-19 disruption on student achievement in Italy. A first relevant contribution of the work relies on exploring students’ results in different educational levels (i.e., primary and middle schools) and subjects. While the literature on the Covid-19 learning loss and, more generally, the evidence on student achievement in Italy tends to focus on reading and mathematics, our paper also provides evidence on English results. This choice has proved to be crucial since the findings highlight different effects associated with English compared to ones of other subjects (reading and mathematics).

We find that school closure produce an increase (even if not statistically significant) in reading results in primary schools – but, by contrast, grade-5 students are associated with a large learning loss in English (especially in reading English). A reverse pattern is found, instead, for grade-8 students, who reported substantial learning loss in mathematics and reading - without showing significant changes in English results. In primary school, the findings regarding reading could be explained by an increase in parental support, especially in advantaged families During school closure, parents could have spent more time at home and had more opportunities to help their children with learning activities and homework. This is particularly true for parents with white-collar jobs that benefitted from larger period of remote working. Parental support is then a plausible mechanism especially for students with higher socioeconomic status, having parents with a higher educational preparation, a job that could be performed online, and more resources to cope with remote learning (e.g., availability of digital tools, a quiet place for studying, fewer domestic duties). On the other hand, contrary to reading and mathematics, the English language may represent a less familiar subject for parents - who are likely to have more difficulties supporting their kids in this discipline. Besides, studying a foreign language requires more social and practical activities that would be difficult to replicate in a remote learning context even with parental support. We should also consider that, during the school year 2020/21, classes have been held face-to-face, but teachers still wore masks. The impossibility of observing teachers’ faces (and the articulation of the words) may have represented a relevant learning disadvantage, especially in the study of foreign languages for younger kids. These factors collectively contribute to explain the substancial learning loss found for English results, which persists even one year after the school closure.

The estimates of the average learning loss in reading and mathematics presented in this study are in line with the findings reported by Borgonovi and Ferrara (2023) and Bazoli et al. (2022), who observe a more severe learning loss for grade-8 students than for grade-5 students, when present. Both studies also confirm the existence of a learning gain in reading for primary school pupils. On the other hand, since this research represents the first investigation of English learning loss in the context of Italian school closures, no comparisons can be made in this regard.

At higher grades, students are more autonomous and less influenced by the support of parents in their schoolwork. Thus, the substantial learning loss in reading and mathematics for grade-8 students may be partially explained by weaker teachers' supervision during online classes compared to face-to-face lessons which is not compensated by parents’ help. In addition, adolescents are likely to suffer more in terms of lack of social interactions and psychological distress (see Cooper et al., 2021; Ellis et al., 2020), and this has in turn a consequence on the academic results (see Dalsgaard et al., 2020). Regarding the lack of effects found for English in grade 8, the higher level of students' autonomy and free time could instead have encouraged a study of the language through non-academic channels, such as watching movies or other media contents in English. Also, the lower learning loss found for listening compared to reading English seems to support this explanation.

The second and most novel contribution of this research concerns the exploration of the between-school variation in learning loss and the factors that contribute to these differences. The empirical results show high heterogeneity in the effects of school closure across schools, especially in primary education. The data provided by teachers through our survey show that these between-school differences are strongly linked to how remote classes were conducted during the lockdown. Digital tools employed by teachers and, especially, the way they assessed their students seem to have influenced academic achievement. A single best practice cannot be identified since the effectiveness of these remote learning techniques is linked to the specific subject and grade. Among the enabling factors to limit the learning loss of students, the results also shed light on the importance of quickly starting online classes after the school closure, and on the role covered by the school principal in supporting the teachers with the organisation of remote teaching.

All the results presented here should be interpreted as a mid-term effect of the Covid-19 school closure. Although data do not allow us to calculate a precise estimate, we can hypothesise that extent of student learning loss right after the school closure was probably larger than the one we estimated one year after. The literature provides evidence of a remarkable recovery of student achievement during the last year of schooling (see Gambi & De Witte, 2021). Also, future research should continue the work presented here by monitoring the evolution of the Covid-19 learning losses over time. It is important to understand how quickly students will recover from the negative consequences produced by the pandemic and which are the best strategies for catching up. Further works may also estimate the effect of Covid-19 school closure on higher educational levels. In the paper, we focus on primary and middle schools since analysing high school education would entail, in the case of Italy, several criticalities. First, Italian high schools were frequently closed during the year 2020/21, with periods of remote classes varying substantially across regions. Second, grade retention is traditionally more frequent than in the lower levels of education, and this phenomenon may cause a bias in the analyses - given that the Italian Ministry of Education suspended the possibility to fail students during the school year 2019/20 (Ordinanza Ministeriale of 16/05/2020). Finally, even if the study of non-compulsory education is crucial, the higher student dropout that characterised high school education may generate a bias in the estimation of learning loss – being impossible to analyse the results of withdrawn students.

This paper opens the path to empirical works on students’ learning loss in Italy and provides relevant results for policymakers. First, we offer novel evidence on how Covid-19 disruption affects academic results, with information on the groups of students and schools that have been most harmed by school closure. Based on this information, the Italian Government could set up specific and targeted strategies to help students to recover the gap in their academic results. Second, the paper offers evidence on the mechanisms explaining the heterogeneity of learning loss and identifies the main determinants and teaching behaviours leading to these differences. Current evidence could be beneficial in providing relevant information on how to better integrate online teaching techniques with traditional learning, in a context (“new normal”) that is going towards higher levels of school digitalisation.

## Ethics statement

The ethics procedure followed the guidelines of the ethics committee of Politecnico di Milano and INVALSI. Consent was obtained from participants.

## Declaration of Competing Interest

None.

## Figures and Tables

**Table A1:** Descriptive statistics of students’ characteristics - Grade 5.

	Obs.		Mean		Std. Dev.		Type	T-test
Variable	Covid-19 cohort	Control cohorts	Covid-19 cohort	Control cohorts	Covid-19 cohort	Control cohorts	range/ type	Ha: diff != 0
female	17,159	33,804	0.491	0.487	0.500	0.500	dummy	ns
postponed	17,159	33,804	0.021	0.025	0.143	0.155	dummy	***
kindergarten	14,116	27,298	0.935	0.933	0.246	0.250	dummy	ns
parent_he	13,443	26,768	0.326	0.294	0.469	0.455	dummy	***
ESCS_stud	16,650	33,020	0.142	0.064	0.909	1.009	[−3.2; 2.4]	***
foreign	16,388	32,236	0.124	0.121	0.330	0.326	dummy	ns
ESCS_class	17,033	33,795	0.135	0.062	0.470	0.540	[−2.5; 1.9]	***
north	17,159	33,804	0.424	0.395	0.494	0.489	dummy	***
centre	17,159	33,804	0.203	0.196	0.402	0.397	dummy	*
south	17,159	33,804	0.373	0.410	0.483	0.492	dummy	***

Notes: *** indicates significance at the 1% level, ** at the 5% level, * at the 10% level and ns: non-significant p-value. P-values refer to the T-test for equal means between control cohort and Covid-19 cohort.

**Table A2:** Descriptive statistics of students’ characteristics - Grade 8.

	Obs.		Mean		Std. Dev.		Type	T-test
Variable	Covid-19 cohort	Control cohorts	Covid-19 cohort	Control cohorts	Covid-19 cohort	Control cohorts	range/ type	Ha: diff != 0
female	14,391	42,080	0.488	0.488	0.500	0.500	dummy	ns
postponed	14,391	42,079	0.057	0.071	0.233	0.258	dummy	***
kindergarten	11,312	33,927	0.934	0.915	0.249	0.279	dummy	***
parent_he	10,960	31,409	0.288	0.267	0.453	0.443	dummy	***
ESCS_stud	14,130	40,668	0.060	0.075	1.019	1.004	[−3.2; 2.1]	ns
foreign	13,692	40,640	0.102	0.099	0.303	0.298	dummy	ns
ESCS_class	14,391	42,074	0.061	0.072	0.417	0.452	[−2.6; 1.5]	***
north	14,391	42,080	0.444	0.407	0.497	0.491	dummy	***
centre	14,391	42,080	0.204	0.210	0.403	0.407	dummy	ns
south	14,391	42,080	0.352	0.383	0.478	0.486	dummy	***

Notes: *** indicates significance at the 1% level, ** at the 5% level, * at the 10% level and ns: non-significant p-value. P-values refer to the T-test for equal means between control cohort and Covid-19 cohort.

**Table A3:** Differences between schools respondent and non-respondent to the survey – Grade 5.

	Obs.		Mean		Std. Dev.		T-test
Variable	Respondents	Non respondents	Respondents	Non respondents	Respondents	Non respondents	Ha: diff != 0
pre-Covid scores (reading)	186	320	0.008	−0.006	0.344	0.402	ns
pre-Covid scores (math)	186	320	−0.036	−0.001	0.404	0.472	ns
pre-Covid scores (english)	186	320	0.007	−0.087	0.403	0.423	ns
% foreign students	183	315	0.142	0.109	0.140	0.120	***
ESCS_school	186	321	0.066	0.116	0.381	0.403	ns
north	186	322	0.516	0.323	0.501	0.468	***
centre	186	322	0.134	0.220	0.342	0.415	**
south	186	322	0.349	0.457	0.478	0.499	**

Notes: *** indicates significance at the 1% level, ** at the 5% level, * at the 10% level and ns: non-significant p-value. The reported p-values are from a T-test for determining whether the means of schools that responded to the survey are significantly different from the means of schools that did not respond.

**Table A4:** Differences between schools respondent and non-respondent to the survey – Grade 8.

	Obs.		Mean		Std. Dev.		T-test
Variable	Respondents	No respondents	Respondents	No respondents	Respondents	No respondents	Ha: diff != 0
pre-Covid scores (reading)	185	204	−0.021	−0.020	0.324	0.377	ns
pre-Covid scores (math)	185	203	−0.046	0.033	0.371	0.385	**
pre-Covid scores (English)	182	192	0.000	−0.044	0.408	0.487	ns
% foreign students	181	201	0.112	0.091	0.116	0.106	*
ESCS_school	185	205	0.037	0.046	0.314	0.346	ns
north	185	205	0.497	0.356	0.501	0.480	***
centre	185	205	0.151	0.215	0.359	0.412	ns
south	185	205	0.351	0.429	0.479	0.496	ns

Notes: *** indicates significance at the 1% level, ** at the 5% level, * at the 10% level and ns: non-significant p-value. The reported p-values are from a T-test for determining whether the means of schools that responded to the survey are significantly different from the means of schools that did not respond.

**Table A5:** Heterogeneity of Covid-19 effect on different groups of students on standardised test scores.

	(1)	(2)	(3)	(4)	(5)	(6)	(7)	(8)
Variables	Reading G5	Reading G8	Mathematics G5	Mathematics G8	English reading G5	English reading G8	English listening G5	English listening G8
COVID	0.014	−0.074***	−0.078***	−0.136***	−0.267***	−0.0304	−0.202***	−0.001
	(0.025)	(0.017)	(0.027)	(0.020)	(0.031)	(0.020)	(0.036)	(0.021)
female	0.194***	0.219***	−0.155***	−0.112***	0.118***	0.191***	0.082***	0.183***
	(0.012)	(0.010)	(0.012)	(0.010)	(0.012)	(0.010)	(0.011)	(0.010)
COVID(=1)#female(=1)	−0.031	−0.021	0.016	−0.073***	−0.072***	−0.017	0.109***	−0.030
	(0.021)	(0.019)	(0.022)	(0.021)	(0.023)	(0.021)	(0.024)	(0.022)
ESCS_stud	0.222***	0.299***	0.202***	0.281***	0.157***	0.302***	0.132***	0.287***
	(0.007)	(0.005)	(0.008)	(0.006)	(0.008)	(0.006)	(0.009)	(0.006)
COVID(=1)#ESCS_stud	0.049***	0.047***	0.069***	0.066***	0.042**	0.057***	0.111***	0.055***
	(0.014)	(0.011)	(0.016)	(0.011)	(0.017)	(0.012)	(0.020)	(0.012)
foreign	−0.318***	−0.334***	−0.220***	−0.133***	0.017	0.084***	0.094***	0.246***
	(0.024)	(0.019)	(0.023)	(0.018)	(0.025)	(0.019)	(0.024)	(0.019)
COVID(=1)#foreign(=1)	0.094**	0.001	0.048	0.018	0.126***	0.071*	0.146***	0.027
	(0.038)	(0.036)	(0.040)	(0.037)	(0.043)	(0.038)	(0.048)	(0.039)
postponed	−0.450***	−0.385***	−0.322***	−0.400***	−0.305***	−0.388***	−0.185***	−0.286***
	(0.042)	(0.020)	(0.041)	(0.019)	(0.047)	(0.022)	(0.045)	(0.022)
kindergarten2	0.078**	0.099***	0.093**	0.078***	−0.010	0.079***	0.018	0.053**
	(0.039)	(0.020)	(0.039)	(0.021)	(0.045)	(0.021)	(0.048)	(0.021)
centre	0.072***	−0.069***	0.088***	−0.122***	−0.045	−0.157***	−0.048	−0.202***
	(0.023)	(0.016)	(0.029)	(0.019)	(0.030)	(0.017)	(0.037)	(0.018)
south	−0.0923***	−0.306***	0.023	−0.490***	−0.114***	−0.482***	−0.251***	−0.665***
	(0.022)	(0.014)	(0.024)	(0.016)	(0.028)	(0.016)	(0.033)	(0.018)
Constant	−0.079*	0.066***	0.020	0.299***	0.090*	0.065***	0.082	0.137***
	(0.041)	(0.029)	(0.041)	(0.023)	(0.049)	(0.023)	(0.052)	(0.023)
Observations	38,131	42,885	38,736	42,833	37,716	42,796	37,698	42,786
R-squared	0.099	0.199	0.076	0.186	0.052	0.189	0.052	0.221

Note: *** *p*<0.01, ** *p*<0.05, * *p*<0.1. Clustered standard errors per class in parentheses. The dependant variables of the eight models are the INVALSI test scores in different grades and subjects. The scores are standardised to have a mean of zero and a standard deviation of one in their respective grades and cohorts. Models estimated using propensity score weights. G5 indicates grade 5 (primary schools), and G8 grade 8 (middle schools).

## Data Availability

The authors do not have permission to share data.

## Footnotes

2 We employed the representative national sample selected by INVALSI in 2020 to merge the standardised test dataset with the survey on teaching remote practices used in the second part of our analysis.

3 We employed the entire population of students in the selected sample of schools as our control group. This approach allows us to obtain a high number of observations, which is crucial for achieving robust matching between the treatment and control groups.

4 The socioeconomic status (ESCS) is calculated by INVALSI by employing a composite indicator that accounts for the occupational status of the parents, their level of education, and the household resources of the family. The indicator is built to have a conventional national mean of 0 and a standard deviation of 1.

5 At least one parent having achieved higher education attainment.

## References

[bib0001] Agasisti, T., & Di Blas, N. (2021). Innovare le scuole con la tecnologia. Indicazioni da una esperienza durante l'emergenza Covid-19. https://re.public.polimi.it/handle/11311/1188065.

[bib0002] Agasisti T., Falzetti P. (2017). Between-classes sorting within schools and test scores: An empirical analysis of Italian junior secondary schools. International Review of Economics.

[bib0003] Ardington C., Wills G., Kotze J. (2021). COVID-19 learning losses: Early grade reading in South Africa. International Journal of Educational Development.

[bib0004] Arenas A., Gortazar L. (2022). ESADE Working Paper.

[bib0005] Bando R., Gallego F., Gertler P., Fonseca D.R. (2017). Books or laptops? The effect of shifting from printed to digital delivery of educational content on learning. Economics of Education Review.

[bib0006] Bazoli N., Marzadro S., Schizzerotto A., Vergolini L. (2022). FBK-IRVAPP Working Papers.

[bib0007] Bertoletti A., Biagi F., Di Pietro G., Karpiński Z. (2023). The effect of the COVID-19 disruption on the gender gap in students’ performance: A cross-country analysis. Large-Scale Assessments in Education.

[bib0008] Bertoletti A., Soncin M., Cannistrà M., Agasisti T. (2023). The educational effects of emergency remote teaching practices—The case of covid-19 school closure in Italy. PloS One.

[bib0010] Birkelund, J.F., .& Karlson, K.B. (.2021). No evidence of a major learning slide 14 months into the COVID-19 pandemic in Denmark, 10.31235/osf.io/md5zn.

[bib0009] Betthäuser B.A., Bach-Mortensen A.M., Engzell P. (2023). A systematic review and meta-analysis of the evidence on learning during the COVID-19 pandemic. Nature Human Behaviour.

[bib0011] Borgonovi F., Ferrara A. (2023). COVID-19 and inequalities in educational achievement in Italy. Research in Social Stratification and Mobility.

[bib0012] Carretero, G.S., Napierala, J., Bessios, A., Magi, E., Pugacewicz, A., Ranieri, M., ... & Gonzalez Vazquez, I. (2021). *What did we learn from schooling practices during the COVID-19 lockdown? Insights from five EU countries* (No. JRC123654). Joint Research Centre (Seville site).

[bib0013] Cascella C. (2020). Intersectional effects of Socioeconomic status, phase and gender on Mathematics achievement. Educational Studies.

[bib0014] Chatterji P., Li Y. (2021). Effects of COVID-19 on school enrollment. Economics of Education Review.

[bib50] Contini D., Di Tommaso M.L., Muratori C., Piazzalunga D., Schiavon L. (2021). The COVID-19 pandemic and school closure: learning loss in mathematics in primary education..

[bib0015] Contini D., Di Tommaso M.L., Muratori C., Piazzalunga D., Schiavon L. (2022). Who lost the most? Mathematics achievement during the COVID-19 pandemic. The BE Journal of Economic Analysis & Policy.

[bib0016] Cooper K., Hards E., Moltrecht B., Reynolds S., Shum A., McElroy E., Loades M. (2021). Loneliness, social relationships, and mental health in adolescents during the COVID-19 pandemic. Journal of Affective Disorders.

[bib0017] Cunha F., Heckman J. (2007). The technology of skill formation. American Economic Review.

[bib0018] Dalsgaard S., McGrath J., Østergaard S.D., Wray N.R., Pedersen C.B., Mortensen P.B., Petersen L. (2020). Association of mental disorder in childhood and adolescence with subsequent educational achievement. JAMA Psychiatry.

[bib0019] Di Pietro G. (2023). The impact of Covid-19 on student achievement: Evidence from a recent meta-analysis. Educational Research Review.

[bib0020] Di Pietro, G., Biagi, F., Costa, P., Karpiński, Z., & Mazza, J. (2020). *The likely impact of COVID-19 on education: Reflections based on the existing literature and recent international datasets* (Vol. *30275*). Luxembourg: Publications Office of the European Union.

[bib0021] Donnelly R., Patrinos H.A. (2021). Learning loss during COVID-19: An early systematic review. Prospects.

[bib0022] Ellis W., Dumas T., Forbes L. (2020). Physically isolated but socially connected: Psychological adjustment and stress among adolescents during the initial COVID-19 crisis. Canadian Journal of Behavioural Science.

[bib0024] European Commission (2020). The Digital Economy and Society Index (DESI). Thematic chapter. Retrieved online from: Https://digital-strategy.ec.europa.eu/en/policies/desi.

[bib0025] Gambi, L., & De Witte, K. (2021). The resiliency of school outcomes after the COVID-19 pandemic. Standardised test scores and inequality one year after long term school closures. FEB Research Report Department of Economics.

[bib0023] Engzell P., Frey A., Verhagen M. (2021). Learning inequality during the Covid-19 pandemic. Proceedings of the National Academy of Sciences.

[bib0026] Giovannella C., Passarelli M., Persico D. (2020). Measuring the effect of the Covid-19 pandemic on the Italian Learning Ecosystems at the steady state: A school teachers’ perspective. Interaction Design and Architecture(s) Journal.

[bib0027] Gore J., Fray L., Miller A., Harris J., Taggart W. (2021). The impact of COVID-19 on student learning in New South Wales primary schools: An empirical study. The Australian Educational Researcher.

[bib0028] Goudeau S., Sanrey C., Stanczak A., Manstead A., Darnon C. (2021). Why lockdown and distance learning during the covid-19 pandemic are likely to increase the social class achievement gap. Nature Human Behaviour.

[bib0029] Haelermans C., Korthals R., Jacobs M., de Leeuw S., Vermeulen S., van Vugt L., de Wolf I. (2022). Sharp increase in inequality in education in times of the COVID-19-pandemic. PloS One.

[bib0030] Hanushek E.A. (1979). Conceptual and empirical issues in the estimation of educational production functions. Journal of Human Resources.

[bib0031] Hevia F.J., Vergara-Lope S., Velásquez-Durán A., Calderón D. (2022). Estimation of the fundamental learning loss and learning poverty related to COVID-19 pandemic in Mexico. International Journal of Educational Development.

[bib0032] INVALSI (2019a). Speciale Rapporto INVALSI 2019. La relazione tra risultati e indicatore ESCS. https://www.invalsiopen.it/rapporto-invalsi-2019-indicatore-escs/.

[bib0033] INVALSI (2019b). Rapporto Prove INVALSI 2019. Rapporto Nazionale. https://invalsi-areaprove.cineca.it/docs/2019/Rapporto_prove_INVALSI_2019.pdf.

[bib0034] Lewis, K., Kuhfeld, M., Ruzek, E., & McEachin, A. (2021). Learning during COVID-19: Reading and math achievement in the 2020-21 school year. *NWEA Brief*.

[bib0035] Lichand G., Doria C.A., Leal-Neto O., Fernandes J.P.C. (2022). The impacts of remote learning in secondary education during the pandemic in Brazil. Nature Human Behaviour.

[bib0036] Maldonado J.E., De Witte K. (2022). The effect of school closures on standardised student test outcomes. British Educational Research Journal.

[bib51] Mushquash A.R., Grassia E. (2022). Coping during COVID-19: examining student stress and depressive symptoms. Journal of American College Health.

[bib0037] OECD (2018).

[bib0038] Patrinos H.A., Vegas E., Carter-Rau R. (2022). (Policy Research Working Paper 10033).

[bib0039] Pier, L., Hough, H.J., Christian, M., Bookman, N., Wilkenfeld, B., & Miller, R. (2021). Covid-19 and the educational equity crisis: Evidence on learning loss from the CORE data collaborative. Policy Analysis for California Education. https://edpolicyinca.org/newsroom/covid-19-and-educational-equity-crisis.

[bib0040] Rodríguez-Planas N. (2022). Hitting where it hurts most: COVID-19 and low-income urban college students. Economics of Education Review.

[bib0041] Schult J., Mahler N., Fauth B., Lindner M.A. (2022). Did students learn less during the COVID-19 pandemic? Reading and mathematics competencies before and after the first pandemic wave. School Effectiveness and School Improvement.

[bib0042] Shanahan L., Steinhoff A., Bechtiger L., Murray A.L., Nivette A., Hepp U. (2022). Emotional distress in young adults during the COVID-19 pandemic: Evidence of risk and resilience from a longitudinal cohort study. Psychological Medicine.

[bib49] SIRD (2020). Per un confronto sulle modalità di didattica a distanza adottate nelle scuole italiane nel periodo di emergenza COVID-19.

[bib0043] Skar G.B.U., Graham S., Huebner A. (2022). Learning loss during the COVID-19 pandemic and the impact of emergency remote instruction on first grade students’ writing: A natural experiment. Journal of Educational Psychology.

[bib0044] Sternadel, D. (2021). The impact of COVID-19 on student learning outcomes across Europe: The challenges of distance education for all. NESET Ad hoc report no. 2/2021.

[bib0045] Tomasik M.J., Helbling L.A., Moser U. (2021). Educational gains of in-person vs. distance learning in primary and secondary schools: A natural experiment during the COVID-19 pandemic school closures in Switzerland. International Journal of Psychology.

[bib48] UNESCO. Coronavirus: School closures [Internet]. 2020 [updated date—2020 August 10]. https://en.unesco.org/themes/education-emergencies/coronavirus-schoolclosures.

[bib0046] Van de Werfhorst H.G. (2021). Inequality in learning is a major concern after school closures. Proceedings of the National Academy of Sciences118.

